# Superpixel-Based Optic Nerve Head Segmentation Method of Fundus Images for Glaucoma Assessment

**DOI:** 10.3390/diagnostics12123210

**Published:** 2022-12-17

**Authors:** Francisco J. Ávila, Juan M. Bueno, Laura Remón

**Affiliations:** 1 Departamento de Física Aplicada, Universidad de Zaragoza, 50009 Zaragoza, Spain; 2 Laboratorio de Óptica, Centro de Investigación en Óptica y Nanofísica, Universidad de Murcia, 30100 Murcia, Spain

**Keywords:** glaucoma, retinal imaging, artificial intelligence, superpixel segmentation

## Abstract

Glaucoma disease is the second leading cause of blindness in the world. This progressive ocular neuropathy is mainly caused by uncontrolled high intraocular pressure. Although there is still no cure, early detection and appropriate treatment can stop the disease progression to low vision and blindness. In the clinical practice, the gold standard used by ophthalmologists for glaucoma diagnosis is fundus retinal imaging, in particular optic nerve head (ONH) subjective/manual examination. In this work, we propose an unsupervised superpixel-based method for the optic nerve head (ONH) segmentation. An automatic algorithm based on linear iterative clustering is used to compute an ellipse fitting for the automatic detection of the ONH contour. The tool has been tested using a public retinal fundus images dataset with medical expert ground truths of the ONH contour and validated with a classified (control vs. glaucoma eyes) database. Results showed that the automatic segmentation method provides similar results in ellipse fitting of the ONH that those obtained from the ground truth experts within the statistical range of inter-observation variability. Our method is a user-friendly available program that provides fast and reliable results for clinicians working on glaucoma screening using retinal fundus images.

## 1. Introduction

Glaucoma is a chronic neurodegenerative disease characterized by the loss of retinal ganglion cells, resulting in distinctive changes in the optic nerve head (ONH) and the retinal nerve fiber layer (RNFL). After cataracts, glaucoma is the second leading cause of blindness in the world. For this reason, early diagnosis is the first step to prevent permanent structural damage and irreversible vision loss [1]. Intraocular pressure (IOP) and standard visual fields are the only end-points accepted by the Food and Drug Administration (FDA) to diagnose glaucoma.

Over the past two decades, objective and quantitative methods such as optical coherence tomography (OCT) [2], scanning laser polarimetry (SLP) [3], and confocal scanning laser confocal scanning laser ophthalmoscopy (CSLO) [4] have been developed to assess both nerve fiber loss and ONH changes produced by glaucoma progression. However, these retinal imaging instruments are often costly and present some drawbacks: CSLO is operator-dependent and therefore prone to inter-observer variabilities, and SLP only provides RNFL data. In addition, visual inspection allows a comprehensive evaluation of the ONH such as optic disc pallor, hemorrhages and vessel tortuosity.

Fundus imaging/photography is the gold standard method used by ophthalmologists to qualitatively assess and evaluate ONH structural changes and to assist in diagnosis of glaucoma [5]. The main advantage of this procedure is its simplicity and cost-effectiveness. However, clinical examination of ONH and RNFL structural changes is subjective and requires qualified experts to classify subjects as normal or glaucomatous.

In addition, there is a considerable intra- and inter-observer variability among qualified specialists when assessing the ONH size. To minimize this effect, different advanced automatic image segmentation techniques have been reported. Glaucoma diagnosis has been aided by automatic detection and segmentation of the ONH, optic disc and vascular tree using morphological techniques [6], adaptive deformable models [7], Hough transform [8], edge and active contour detection [9,10], local fitting and probability active shape models [11,12], deformable model approach [13], K-means clustering [14] and intensity inhomogeneity analysis [15].

Retinal segmentation results have been noticeably improved with the irruption of artificial intelligence algorithms providing deep learning analysis through convolutional neural networks (CNNs) [16]. Those recent computer-vision algorithms provide not only objects’ detection within fundus images, but accurate segmentation of the optic disc and glaucoma classification [17,18,19]. Simple Linear Iterative Clustering (SLIC) has also previously reported for automated glaucoma screening [20].

The present study presents an automatic method based on superpixel (SP) classification by extracting structural information from high-quality RGB fundus images, using statistical pixel level and then classifying the features into a Support Vector Machine supervised learning model. In comparison to the previous published method, here we develop a user-friendly unsupervised version of an automatic SP-based tool for fast segmentation of the optic disc from fundus images.

## 2. Materials and Methods

### 2.1. Retinal Fundus Dataset

The DRIONS-DB retinal image public database [21] was employed for the performance evaluation of the proposed algorithm. The database consists of 110 color digital fundus retinal images from Caucasian patients presenting chronic glaucoma (23.1%) and eye hypertension (79.6%). The mean age of the patients was 53 years old (±13 standard deviation). The DRIONS-DB dataset also includes the ground truth of two experienced medical experts. The ONH contours were stored as X-Y spatial coordinates corresponding to 36 sequenced points. In our work, we used the averaged contour of those two experts.

**Figure 1:**
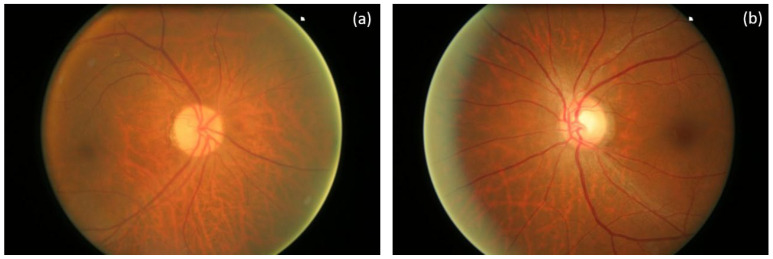
Examples of fundus images from a healthy (**a**) and glaucomatous eye (**b**) obtained from the *ORIGA* dataset [22].

Moreover, 605 retinal fundus images from non-glaucomatous (168 images) and glaucomatous eyes (482 images) from the ORIGA (-light) retinal fundus image database [22] were employed to test and validate the capabilities of the proposed algorithm to discriminate both types of retinas. ORIGA (-light) images were marked by experts from the Singapore Malay Eye Study [22]. No sex differences were found in both databases. Figure 1 shows examples of fundus images from ORIGA database corresponding to a control and a glaucomatous eye.

### 2.2. Algorithm Description and Image Processing

Given an image, the SP segmentation technique [23] groups the pixels with similar color or grayscale levels and structural (texture) properties. In other words, the algorithm sorts structurally similar pixels of an image to create meaningful segments or clusters that are sensitive to low-level properties.

**Figure 2:**
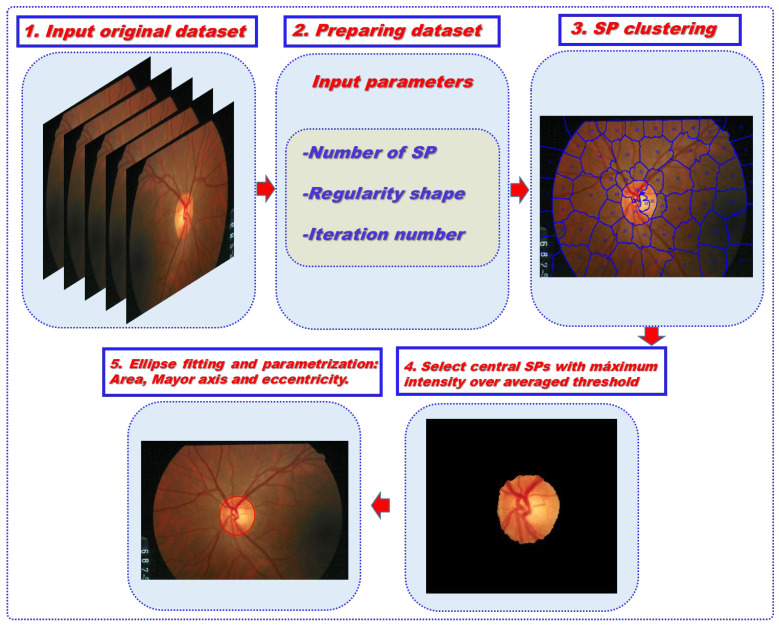
Schematic diagram of the sequential procedure of the algorithm used for automatic ONH segmentation herein.

In this work, a custom script has been written in Matlab^TM^ using the main Matlab function “superpixels” based on a linear iterative clustering algorithm with three input arguments: number of SP to be detected, number of iterations within the process and irregularity rate of SPs. The program is based on an automatic 5-step procedure as shown in Figure 2. Once the set of RGB images are automatically loaded (step #1), the operator sets the initial parameterization by selecting the maximum number of SPs to be detected, the regularity shape rate and the iteration number of the process (step #2). Each image is clustered and the detected SPs numbered (step #3). The program calculates the mean intensity of each SP and scans the clustered image searching for those SPs showing significantly higher pixel intensity than the global average (step #4). From those detected SPs, the program binarizes the image and calls the “bwboundaries” Matlab function for tracing the contour boundaries, which returns a coordinates matrix that is employed for ellipse fitting using the least squares criterion (Step #5).

**Figure 3:**
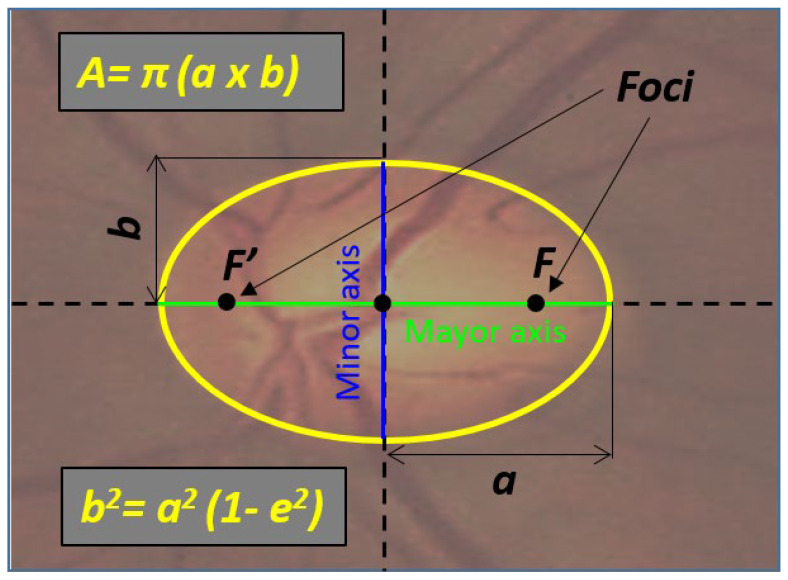
Representation of the computed ellipse parameters. (A) Area of the ellipse; (e) eccentricity; (a) semi-major axis; (b) semi-minor axis; (F′) and (F) focus points.

If an ellipse cannot be found (but a parabola or hyperbola), then, an empty structure is returned. If the ellipse can be fitted, then the axes of the ellipse (*a*), area (A) and eccentricity (*e*) are computed as schematized in Figure 3.

On the other hand, the same procedure used to fit the ONH contour to an ellipse was also applied to average the X-Y coordinates annotated by the two experts in order to compare our automatic detection with the ground truth.

### 2.3. Data Analysis

Statistical analysis and graphic representation were performed in Sigmaplot 14. 0 scietific software (Systat Software Inc., Chicago, IL, USA).

**Figure 4:**
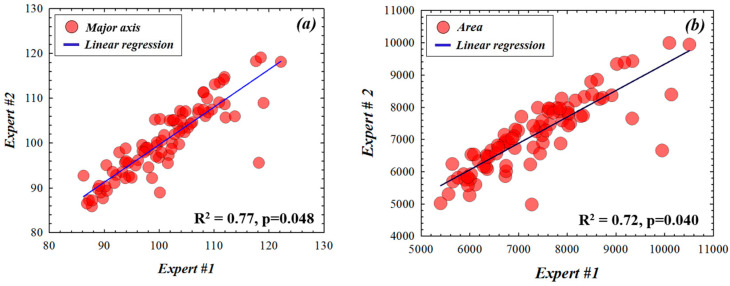
Comparisons of major axis (**a**) and area (**b**) values of the ONH ellipse fitting drawn by the two experts. Significance level: *p* = 0.05.

**Figure 5:**
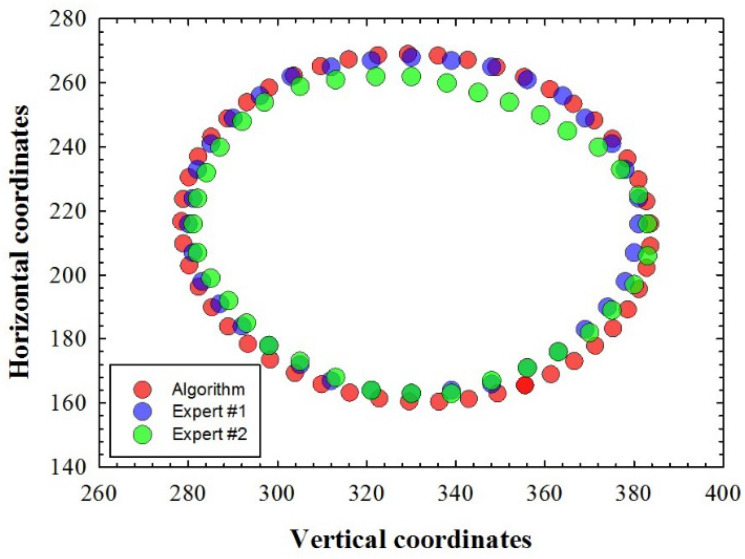
X-Y coordinates of the ONH contour detected by our algorithm (red dots) and those traced by the experts (blue and green dots).

**Figure 6:**
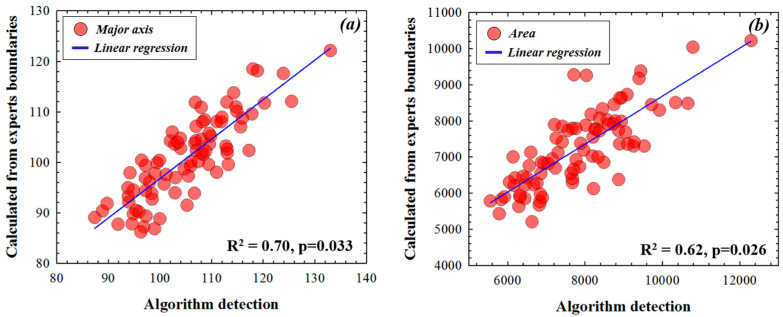
Comparison of the OHN fitting ellipse parameters (major axis (**a**), total area (**b**)) computed by the automatic segmentation algorithm and the mean of those provided by the experts. Significance level: *p* = 0.05.

Data shown in Figure 4, Figure 5 and Figure 6 correspond to the computed output values from the expert boundaries, the coordinates obtained by the experts compared to the algorithm and the expert boundaries outputs compared to the algorithm detection, respectively. The analysis of the data consisted of Spearman’s correlation and linear regression statistical analysis. The significance of the regressions was indicated by the *p*-value (significance level, *p* < 0.05).

**Figure 7:**
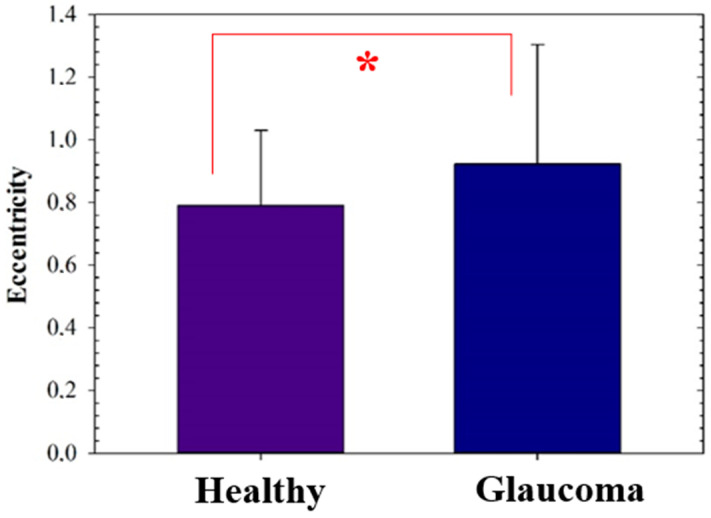
Ellipse eccentricity values computed from healthy and glaucoma eyes. Values were statistically different (*p*-value (*) = 0.014). Significance level *p* = 0.05. Error bars show the standard deviation of the means.

Data shown in Figure 7 corresponds to the mean (±standard deviation) values of the eccentricity computed values for each group (i.e., healthy and glaucomatous groups). Statistical t-test analysis was employed to compare both groups.

## 3. Results

Figure 4 compares the inter-observer variability between the two glaucoma experts for the total area and the major axis of each ellipse contouring the ONH for all retinal fundus images involved. Although there exist discrepancies, the boundaries traced by these clinicians are statistically correlated (R^2^ = 0.77, *p* = 0.048 and R^2^ = 0.72, *p* = 0.040 for major axis and area, respectively).

As an example, for a randomly chosen fundus image of the dataset, Figure 5 presents the X-Y coordinates marked by the two ophthalmologists (blue and green symbols) and the coordinates of the ONH contour detected using the unsupervised algorithm here developed (red symbols). As expected, the plot shows some inter-observer variability, as well as differences between the results from the two experts and those obtained using our automatic detection method. However, a statistical analysis (*t*-test) revealed neither significant differences between the two experts, nor between the experts and the automatic detection.

Figure 6 depicts the comparison of the two parameters of the ellipse used here (area and major axis length) between the automatic detection and the averaged values from the two experts. Spearman’s correlation revealed similar discrepancies between automatic detection and the average of the two experts. Correlation values for major axis and area of the ellipse were 0.70 (*p* = 0.033) and 0.62 (*p* = 0.026), respectively. According to the correlation values presented in Figure 4, our finding proves that the values obtained with our algorithm are consistent with the inter-observer variability observed between the two experienced clinicians.

Finally, since the ONH segmentation procedure is an algorithm working under an unsupervised modality (i.e., no training process needed), it requires validation in terms of glaucoma screening. For this aim, the ORIGA [22] database above mentioned was employed to compute the eccentricity of ellipse fitted from the superpixel segmentation of the ONH in both healthy (N = 168 patients) and glaucomatous eyes (N = 482 patients). Figure 7 compares the results obtained. The statistical analysis (t-test analysis) revealed significant differences between the two groups (*p* = 0.014).

## 4. Discussion and Conclusions

Machine Learning algorithms have provided valuable support in Ophthalmology. Advanced retinal segmentation tools have been used in retinal fundus photography, scanning confocal microscopy imaging and OCT [24,25]. However, obtaining the required data volume, preprocessing for feature extraction and computational costs for the training step may become critical in those cases when the timeline plays against the need of a fast diagnosis.

In this work, we present an automatic method based on superpixel (SP) classification by extracting structural information from high-quality RGB fundus images. To evaluate the proposed method, we use two public databases: DRIONS-DB [21] and ORIGA(-light) [22]. DRIONS-DB retinal image public data were employed for the performance evaluation of the proposed algorithm, the quantitative evaluation was carried out by measurement of the total area and the major axis of each ellipse contouring the ONH for all retinal fundus images. ORIGA(-light) [22] retinal image public data were employed to test and validate the capabilities of the proposed algorithm to discriminate between non-glaucomatous and glaucomatous eyes. For that, we compute the eccentricity of ellipse fitted from the superpixel segmentation of the ONH in both groups.

Previously to our work, some automatic retinal fundus image segmentation methods have been published, including active shape models [26] or region of interest classification methods [27,28].

In particular, SP segmentation methodology has aroused great interest in the development of deep learning algorithms for its application in large image databases [29]. SP segmentation methodology has been previously reported for optic cup segmentation for glaucoma screening. Xu et al. [30] developed a classification learning framework for automatic localization of the optic cup based on the SP segmentation concept. This method was later improved by an unsupervised SP segmentation approach based on an adaptive low-rank representation [31].

The study by Cheng et al. [32] employed the ORIGA [22] dataset to extract features from optic disc and cup to classify between healthy and glaucomatous eyes using the support vector machines library. This database has also been employed to validate the methods proposed herein.

In this sense, this work goes a step further and deals with an automatic unsupervised machine learning method that uses a fast digital SP ONH segmentation for glaucoma screening. This is an easy-to-use tool, where the operator does not require any programming skills (only to set the segmentation sensitivity once the image is loaded). From the segmentation process, different parameters of the best ONH fitting ellipse were used, such as the major axis, the area and the eccentricity. The *DRIONS-DB* retinal fundus images database was used to test the method and measurements obtained through this segmentation and were compared to those provided by two different ophthalmologists. Currently, there is clinical evidence suggesting that structural changes (e.g., optic nerve measured using imaging technologies) were detected earlier than functional changes (e.g., visual field abnormalities) in glaucoma assessment [33]. Our work focused on structural analysis of retinal fundus images and the results showed that the proposed algorithm provided similar ONH objective parameters (major axis and area) to those obtained from experienced medical experts (see Figure 4, Figure 5 and Figure 6). The experimental error of the proposed method also correlates with that obtained from the inter-observer variability.

In the early 1970s, Weisman et al. [33] reported vertical elongation of the ONH as a consequence of glaucoma progression; since then some studies on morphometric analysis of ONH imaging have revealed vertical elongation of the optic cup [34] and Bruch´s membrane deformation [35] as a consequence of glaucoma damage. In that sense, our findings on computed eccentricity were in agreement with those previous findings, corroborating that ONH elongation is associated with glaucoma damage, that is, glaucoma eyes showed a significantly higher eccentricity value than healthy eyes (Figure 7).

In conclusion, our proposed method proves that unsupervised segmentation may constitute a complementary clinical powerful tool for an objective glaucoma screening and to classify between healthy and glaucomatous eyes. The procedure will be of great interest when high computational costs, large datasets and training processing are not available. Future work will include the incorporation of new capabilities of the algorithm for sub-classification of glaucomatous eyes.

## Data Availability

Data underlying the results presented in this paper are available in Databases 1 and 2. References: [21,22].

## References

[1] Remo S., De Moraes C.G., Cioffi G.A., Ritch R. (2015). Why do people (still) go blind from Glaucoma?. Trans. Vis. Sci. Technol..

[2] Bussel I., Wollstein G., Schuman J. (2013). OCT for glaucoma diagnosis, screening and detection of glaucoma progression. Br. J. Ophthalmol..

[3] Lemij H.G., Reus N.J. (2008). New developments in scanning laser polarimetry for glaucoma. Curr. Opin. Opthalmol..

[4] Yaghoubi M., Moradi-Lakeh M., Mokhtari-Payam M., Fakhraie G., Shokraneh F. (2015). Confocal scan laser ophthalmoscope for diagnosing glaucoma: A systematic review and meta-analysis. Asia Pac. J. Ophthalmol..

[5] Chakrabarti L., Joshi G.D., Chakrabarti A., Raman G.V., Krishnadas S.R., Sivaswamy J. (2016). Automated Detection of Glaucoma from Topographic Features of the Optic Nerve Head in Color Fundus Photographs. J. Glaucoma.

[6] Walter T., Klein J.C. (2001). Segmentation of color fundus images of the human retina: Detection of the optic disc and the vascular tree using morphological techniques. Proceedings of the Second International Symposium on Medical Data Analysis.

[7] Haleem M.S., Han L., Hemert J.V., Li B., Fleming A., Pasquale L.R., Song B.J. (2018). A novel adaptive deformable model for automated optic disc and cup segmentation to aid glaucoma diagnosis. J. Med. Syst..

[8] Zhu X., Rangayyan R.M. Detection of the optic disc in images of the retina using the hough transform. Proceedings of the International Conference of the IEEE Engineering in Medicine and Biology Society.

[9] Aquino A., Gegúndez-Arias M.E., Marín D. (2010). Detecting the optic disc boundary in digital fundus images using morphological, edge detection, and feature extraction techniques. IEEE Trans. Med. Imaging.

[10] Chan T., Vese L. (2002). An active contour model without edges. IEEE Trans. Image Process..

[11] Tang L., Garvin M.K., Kwon Y.H., Abramoff M.D. (2012). Segmentation of optic nerve head rim in color fundus photographs by probability based active shape model. Investig. Ophthalmol. Vis. Sci..

[12] Gao Y., Yu X., Wu C., Zhou W., Lei X., Zhuang Y. (2019). Automatic optic disc segmentation based on modified local image fitting model with shape prior information. J. Healthc. Eng..

[13] Xu J., Chutatape O., Sung E., Zheng C., Kuan P.C.T. (2007). Optic disk feature extraction via modified deformable model technique for glaucoma analysis. Pattern Recognit..

[14] Ayub J., Ahmad J., Muhammad J., Aziz L., Ayub S., Akram U., Basit I. Glaucoma detection through optic disc and cup segmentation using K-mean clustering. Proceedings of the 2016 International Conference on Computing, Electronic and Electrical Engineering (ICE Cube).

[15] Zhang K., Zhang L., Lam K., Zhang D. (2016). A level set approach to image segmentation with intensity inhomogeneity. IEEE Trans. Cybern..

[16] Fu H., Cheng J., Xu Y., Liu J., Lu L., Wang X., Carneiro G., Yang L. (2019). Glaucoma Detection Based on Deep Learning Network in Fundus Image. Deep Learning and Convolutional Neural Networks for Medical Imaging and Clinical Informatics.

[17] Park K., Kim J., Lee J. (2020). Automatic optic nerve head localization and cup-to-disc ratio detection using state-of-the-art deep-learning architectures. Sci. Rep..

[18] Sreng S., Maneerat N., Hamamoto K., Win K. (2020). Deep Learning for Optic Disc Segmentation andGlaucoma Diagnosis on Retinal Images. Appl. Sci..

[19] Gheisari S., Shariflou S., Phu J., Kennedy P.J., Agar A., Kalloniatis M., Golzan S.M. (2021). A combined convolutional and recurrent neural network for enhanced glaucoma detection. Sci. Rep..

[20] Mohamed N.A., Zulkifley M.A., Zaki W.M., Hussain A. (2019). An automated glaucoma screening system using cup-to-disc ratio via Simple Linear Iterative Clustering superpixel approach. Biomed. Signal Process. Control..

[21] Carmona E.J., Rincón M., García-Feijoo J., Martínez-de-la-Casa J.M. (2008). Identification of the optic nerve head with genetic algorithms. Artif. Intell. Med..

[22] Zhang Z., Yin F., Liu J., Wong W.K., Tan N.M., Lee B.H., Cheng J., Wong T.Y. (2010). ORIGA(-light): An online retinal fundus image database for glaucoma analysis and research. Annu. Int. Conf. IEEE Engin. Med. Biol. Soc..

[23] Ren X., Malik J. Learning a classification model for segmentation. Proceedings of the Ninth IEEE International Conference on Computer Vision.

[24] Masumoto H., Tabuchi H., Nakakura S., Naofumi I. (2018). Deep-learning Classifier with an Ultrawide-field Scanning Laser Ophthalmoscope Detects Glaucoma Visual Field Severity. J. Glaucoma.

[25] Ran A., Tham C., Chan P., Cheng C.-Y., Tham Y.-C., Rim T.H., Cheung C.Y. (2021). Deep learning in glaucoma with optical coherence tomography: A review. Eye.

[26] Yin F., Liu J., Ong S., Sun Y., Wong D.W.K., Tan N.M., Cheung C., Baskaran M., Aung T., Wong T.Y. (2011). Model-based optic nerve head segmentation on retinal fundus images. IEEE Int. Conf. Eng. Med. Biol. Soc..

[27] Wong D., Lim J., Tan N., Tan N.M., Zhang Z., Lu S., Li H., Teo M.H., Chan K.L., Wong T.Y. (2009). Intelligent fusing of cup-to-disc ratio determination methods for glaucoma diagnosis. Int. Conf. Engin. Med. Biol. Soc..

[28] Xu Y., Xu D., Lin S., Liu J., Cheng J., Cheung C., Aung T., Wong T.Y. (2011). Sliding window and regression based cup detection in digital fundus images for glaucoma diagnosis. Med. Image Comput. Comput. Assist. Interv..

[29] Tan N., Xu Y., Goh W., Liu J. (2015). Robust multi-scale superpixel classification for optic cup localization. Comput. Med. Imaging Graph.

[30] Xu Y., Liu J., Lin S., Xu D., Cheung C.Y., Aung T., Wong T.Y., Ayache N., Delingette H., Golland P., Mori K. (2012). Efficient Optic Cup Detection from Intra-image Learning with Retinal Structure Prior. Medical Image Computing and Computer-Assisted Intervention—MICCAI 2012.

[31] Xu Y., Duan L., Lin S., Chen X., Wong D.W.K., Wong T.Y., Liu J. (2014). Optic cup segmentation for glaucoma detection using low-rank superpixel representation. Med. Image Comput. Comput. Assist. Interv..

[32] Cheng J., Liu J., Xu J., Yin F., Wong D.W.K., Tan N.-M., Tao D., Cheng C.-Y., Aung T., Wong T.Y. (2013). Superpixel classification based optic disc and optic cup segmentation for glaucoma screening. IEEE Trans. Med. Imaging.

[33] Weismann R.L., Asseff C.F., Phelps C.D., Podos S.M., Becker B. (1973). Vertical elongation of the optic cup in glaucoma. Trans. Am. Acad. Ophthalmol. Otolaryngol..

[34] Mohammadzadeh V., Rabiolo A., Fu Q., Morales E., Coleman A.L., Law S.K., Caprioli J., Nouri-Mahdavi K. (2020). Longitudinal macular structure-function relationship in glaucoma. Ophtalmology.

[35] Lee S., Han S., Young M., Beg M.F., Sarunic M.V., MacKenzie P.J. (2014). Optic Nerve Head and Peripapillary Morphometrics in Myopic Glaucoma. Glaucoma. Investig. Opthalmol. Vis. Sci..

